# Transabdominal Laparoscopic Repair of Amyand's Hernia: A Case Report

**DOI:** 10.1155/2011/823936

**Published:** 2011-09-07

**Authors:** Bachir Elias, Elie Chelala, Jean-Louis Allé

**Affiliations:** ^1^General Surgery Department, University Hospital of Tivoli, Avenue Max Buset 34, 7100 La Louvière, Belgium; ^2^General Surgery Department, Bel-Air Hospital, 1-3, rue de Friscaty, 57126, Thionville, France

## Abstract

The occurrence of appendicitis within an inguinal hernia, known as Amyand's hernia, is a rare condition. Laparoscopic approach to this type of hernia is not widely used, and there is little data comparing it to the open surgery approach. This article presents a case of Amyand's hernia treated successfully by laparoscopy via a transabdominal approach supported by a short review of literature on this rare condition.

## 1. Introduction

In 1735, Claudius Amyand, surgeon to King Georges II, performed the first recorded appendectomy for a perforated appendicitis within the inguinal canal; thus, Amyand's hernia became the term to describe appendicitis, a perforated appendix [1], or a noninflamed appendix [2] within an irreducible inguinal hernia. The incidence of the latter form is estimated to be 1% of adult inguinal hernia repair, but the reported incidence of appendicitis in the inguinal sac is rarer and ranges between 0,08 to 0,13% [3].

## 2. Case Report

A 72-year-old female with no significant past medical or surgical history came to our emergency room complaining of a day-old right inguinal pain irradiating to the right iliac fossa. The pain was increasing in intensity without any associated gastrointestinal or genitourinary symptoms. A physical exam showed normal vital signs and an irreducible tender right inguinal mass. The abdomen was soft, and an uncomplicated umbilical hernia was noted.

**Figure 1:**
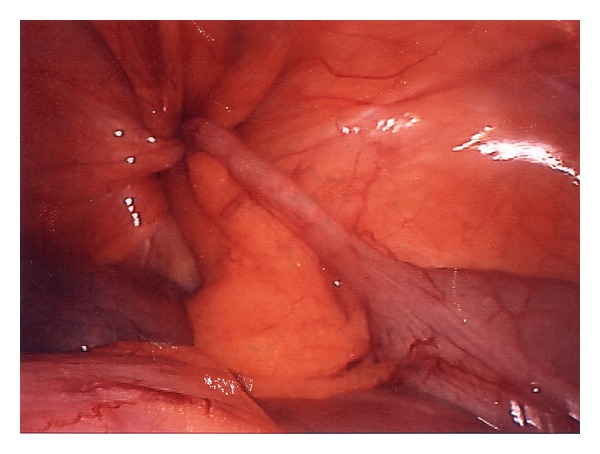
Laparoscopic view of Amyand's hernia. Appendix incarcerated within an indirect inguinal hernia.

Routine laboratory investigations yielded normal results. An ultrasound was performed upon which a right inguinal sac containing fluid and an enlarged inflamed appendix of 7 mm of diameter was diagnosed. A transabdominal laparoscopic approach confirmed the diagnosis of appendicitis incarcerated with a large lipoma within an indirect inguinal hernia (Figure 1).

After reduction, the distal end of the appendix was found necrotic; we performed an appendectomy, an excision of the lipoma, a reduction and closure of the hernial sac with nonabsorbable suture without any prosthetic reinforcement.

The postoperative course was uneventful, and histopathology confirmed the diagnosis of acute appendicitis and benign lipoma.

The patient was admitted 3 months later for elective laparoscopic mesh repair of her umbilical hernia without evidence of recurrent right inguinal hernia.

## 3. Discussion

Amyand's hernia was reported in neonates as in elderly patient with a slight male predominance [4].

The real pathophysiology of Amyand's hernia is not clear. Some authors argued that the appendix becomes inflamed and enters the inguinal sac which leads to adhesions and bacterial overgrowth. A contraction or sudden increase in intra-abdominal pressure may cause further incarceration of the appendix and further inflammation [5].

Others theories suggest that the uninflamed appendix enters the inguinal sac and becomes incarcerated and then inflamed [1].

In our case, it is probable that the presence of a large lipoma could be a predisposing factor to further incarceration of the appendix and its secondary inflammation.

The clinical presentation of Amyand's hernia is usually that of an incarcerated hernia, but the pain tends to be episodic and cramp-like instead of a constant dull ache seen in a strangulated bowel. Mac Burney sign, leukocytosis, and fever usually are uncommon because of the sequestrated nature of the inflammatory process [6].

The diagnosis of Amyand's hernia can be difficult based only on a clinical setting. Apostolidis et al. [1] reported only one case of Amyand's hernia diagnosed preoperatively out of 60 cases between 1959 and 1999; but with the evolution of radiological investigations, Amyand's hernia can be properly diagnosed in a preoperative setting by ultrasound as in our case, and/or by CT scan [7, 8]. We believe that in some equivocal cases a laparoscopic approach can confirm the diagnosis and serve as a therapeutic tool at the same time.

The treatment of Amyand's hernia and the surgical approach depend mainly on the inflammatory status of the appendix [2]. Traditional treatment is an appendectomy through an inguinal incision followed by a repair of the hernia defect [3], or through a lower midline celiotomy in case of abscess and for a better exploration of the abdominal cavity [4]. Laparoscopic management can be more advantageous taking into consideration its role in diagnosing or confirming Amyand's hernia, in exploring the abdominal cavity for other pathologies or complicated hernia, and in being a therapeutic tool at the same time. Laparoscopic appendectomy followed by open repair of the hernia is a choice [9], but a total laparoscopic approach either preperitoneal [10], or as in our case transabdominal, is feasible and safe especially if the hernial sac can be reduced and closed. Comparing laparoscopic approach to open approach, in terms of long-term results and complications, can be difficult considering the rarity of this form of hernia.

The use of prosthetic material in repairing Amyand's hernia is still controversial, but most authors do not recommend hernia repair with mesh [3, 9] except in selected patients with an uninflamed appendix. Even thus, incidental appendectomy in case of a normal appendix is not recommended [2].

## 4. Conclusion

Amyand's hernia is a rare condition and should be considered in the differential diagnosis of incarcerated inguinal hernia, with an acute surgical management. 

Appendectomy and primary hernia repair at the same time can be safely done by laparoscopy.

The use of synthetic mesh remains controversial and depends mainly of the inflammatory aspect of the appendix.
